# Medial joint line obliquity and medial meniscus tear were associated with medial meniscal extrusion: A case–control study

**DOI:** 10.1002/jeo2.70584

**Published:** 2025-12-07

**Authors:** Kazuki Asai, Yosuke Shima, Kenichi Goshima, Kazunari Kuroda, Takeshi Oshima, Yasushi Takata, Mitsuhiro Kimura, Kengo Shimozaki, Tomoyuki Kanayama, Naoki Takemoto, Manase Nishimura, Kentaro Fujita, Satoru Demura, Junsuke Nakase

**Affiliations:** ^1^ Department of Orthopedic Surgery Keiju Medical Center Nanao Japan; ^2^ Department of Orthopedic Surgery Graduate School of Medical Science Kanazawa University Kanazawa Japan; ^3^ Department of Orthopedic Surgery KKR Hokuriku Hospital Kanazawa Japan; ^4^ Department of Orthopedic Surgery Kanazawa Munehiro Hospital Kanazawa Japan; ^5^ Department of Orthopedic Surgery Yawata Medical Center Komatsu Japan; ^6^ Department of Orthopedic Surgery Kanazawa Municipal Hospital Kanazawa Japan; ^7^ Department of Orthopedic Surgery Fukui General Hospital Fukui Japan; ^8^ Department of Orthopedic Surgery Ishikawa Prefectural Central Hospital Kanazawa Japan; ^9^ Department of Orthopedic Surgery Suzu general Hospital Suzu Japan

**Keywords:** early‐stage knee osteoarthritis, knee joint, medial joint line obliquity, medial meniscal extrusion, meniscus tear

## Abstract

**Purpose:**

Medial meniscal extrusion is a key factor in accelerating knee osteoarthritis. Medial joint line obliquity causes increased contact pressure on the medial meniscus, which potentially induces medial meniscal extrusion. Our hypothesis was that medial joint line obliquity contributes to an increase in medial meniscal extrusion in both supine and upright position during early‐stage knee osteoarthritis (KOA).

**Methods:**

We analysed 124 knees with early‐stage KOA. Medial joint line obliquity (knee joint line obliquity ≤ − 3°) and posterior tibial slope ( ≥ 6°) were assessed using standing full‐length lower extremity radiography and magnetic resonance imaging, respectively. Medial meniscal extrusion was measured using ultrasonography in the supine and upright positions. Cases with excessive posterior tibial slope were excluded to minimise the influence of the posterior tibial slope, and the remaining 89 knees were divided into medial joint line obliquity (*n* = 45; age = 58.3 ± 10.5 years) and control groups (*n* = 44; age = 59.8 ± 11.9 years). Each measurement was compared between the two groups using t‐test or chi‐square test. Multiple regression analysis was performed to determine the independent factor for medial meniscal extrusion.

**Results:**

The medial joint line obliquity group showed significantly higher medial meniscal extrusion than the control group (supine, 3.0 ± 1.0 vs. 2.4 ± 0.9 mm, *p* = 0.004; upright, 3.7 ± 1.0 vs. 3.2 ± 1.1 mm, *p* = 0.023). Meniscal tear was present in 75.6% and 75% of the medial joint line obliquity and control groups, respectively, with no significant difference in tear type distribution (*p* = 0.999). Medial joint line obliquity and medial meniscus tear were independent factors for increased medial meniscal extrusion in both the supine (*p* = 0.002 and *p* = 0.003, respectively) and upright positions (*p* = 0.017 and *p* = 0.001, respectively).

**Conclusion:**

Medial joint line obliquity and medial meniscus tear may be risk factors for medial meniscal extrusion in both the supine and upright positions in early‐stage KOA.

**Level of Evidence:**

Level IV.

**Abbreviations**
HKA: hip‐knee‐ankle angle
ICC: intraclass correlation coefficient
KJLO: knee joint line obliquity
KOA: knee osteoarthritis
K/L: Kellgren–Lawrence
LDFA: lateral distal femoral angle
MCL: medial collateral ligament
MJLO: medial joint line oblique
MME: medial meniscal extrusion
MMPRT: medial meniscal posterior root tear
MPTA: medial proximal tibial angle
MRI: magnetic resonance imaging
PTS: posterior tibial slope

## INTRODUCTION

Early‐stage knee osteoarthritis (KOA) is primarily characterised by Kellgren–Lawrence (K/L) Grade 0 or 1, accompanied by knee joint pain [9, 11]. This stage represents the initial phase of the disease, where structural changes are minimal but symptomatic discomfort begins to manifest. Some of the key pathological features associated with early‐stage KOA are medial meniscal extrusion (MME) and medial meniscal posterior root tear (MMPRT) [19]. Notably, MME is considered a critical factor in accelerating KOA progression by increasing load‐bearing stress on the knee joint [5]. MME occurs when the circumferential fibres of the menisci fail to effectively redistribute compressive axial loads, known as hoop stresses, into tensile forces due to meniscal tear or degeneration [5]. Despite extensive studies, the specific factors that exacerbate MME remain unclear, highlighting the need for further investigation.

Medial joint line obliquity (MJLO) can induce medial femoral condyle subluxation on the tibial plateau medially and create a shearing force, leading to increased contact pressure on the medial meniscus, particularly in its middle portion [22]. Previous studies have highlighted a correlation between a steep posterior tibial slope (PTS) and MMPRT [4, 6]. A higher PTS increases the force on the posterior medial meniscal root, leading to MMPRT, which results in significant MME [3, 12, 21]. However, studies evaluating the correlation between MME and MJLO are scarce.

To evaluate the effect of MJLO on MME, the influence of the PTS should be minimised. When evaluating lower limb alignment, it is important not only to assess varus and valgus deformities but also to understand the biomechanical impact of joint line obliquity on the knee joint, particularly on MME, in order to consider appropriate treatment strategies for early‐stage KOA. Therefore, this study aimed to evaluate the effects of MJLO on MME on early‐stage KOA. Our hypothesis was that MJLO contributes to an increase in MME in both supine and upright position during early‐stage KOA.

## METHODS

**Figure 1:**
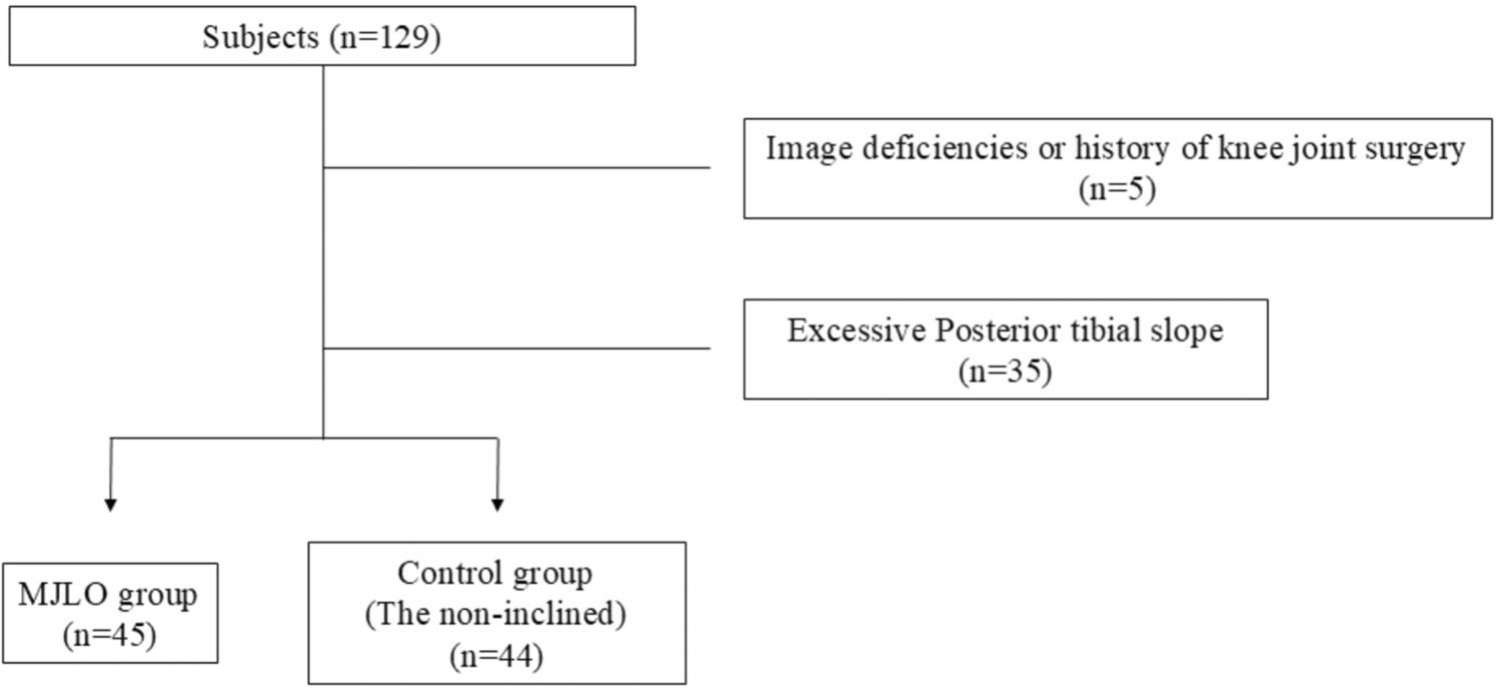
Flowchart of the study. MJLO, medial joint line obliquity.

A total of 129 knees from 125 patients (4 patients had both knees analysed) diagnosed as early‐stage KOA were included in this study. Ultrasonographic, magnetic resonance imaging (MRI), and radiographic data were obtained from eight facilities and measured by two investigators (K.A. and N.T.). Early‐stage KOA was defined as medial knee pain with a K/L grade of 0 or 1 on radiographs [13]. The inclusion criterion was diagnosis of early‐stage KOA without pain in other joints. Those with imaging deficiencies (*n* = 3) and history of knee joint surgery (*n* = 2) were excluded. Finally, 124 knees from 120 patients were included in this study. Monthly meetings were held to standardise ultrasonographic imaging and share study progress across 8 facilities. Ultrasonographic images were used to measure MME in both the supine and upright positions, full‐length standing radiographs were used to determine the alignment of the lower limbs, and MRIs were used to determine the medial tibial posterior slope and meniscal degenerative tears. Then, those with excessive PTS (*n* = 35), defined as PTS of medial tibial condyle ≥ 6°, were excluded to minimise the influence of the PTS, and the remaining 89 knees were divided into medial joint line obliquity (*n* = 45) and control groups (*n* = 44). A flowchart of this study is presented in Figure 1. This study was approved by the ethics committee of our hospital (approval number 2328), and written informed consent was obtained from each patient.

### Radiographic assessment

**Figure 2:**
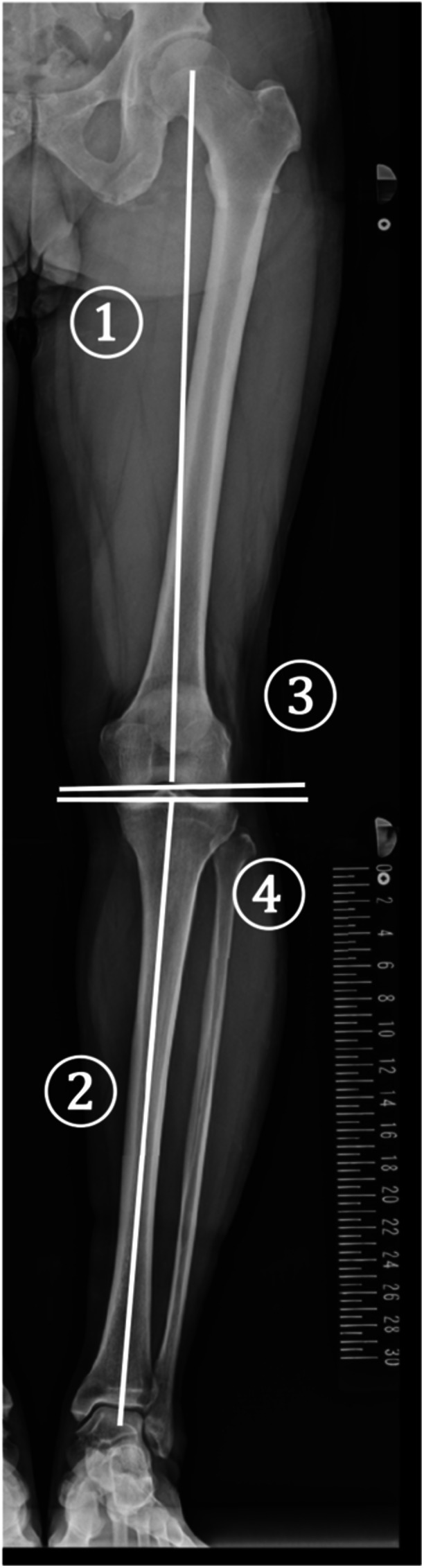
Radiographic assessment. The standing full‐length lower extremity was used for the measument. Each of the lines was drawn as follows: (1) Mechanical axis of the femur. (2) Mechanical axis of the tibia. (3) Tangent to the femoral condyle. (4) Tangent to the tibial plateau. The hip–knee–ankle angle (HKA), lateral distal femoral angle (LDFA), and medial proximal tibial angle (MPTA) correspond to the angles defined by lines (1) and (2), (1) and (3), and (2) and (4), respectively. Knee joint line obliquity (KJLO) corresponds to the angle defined by line (4) and the horizontal line.

All patients underwent anteroposterior radiographic imaging of the standing full‐length lower extremities. Images were captured on one side at a time, and only images of the affected side were used in the analysis. Lower limb deformity indices, including the hip‐knee‐ankle angle (HKA), lateral distal femoral angle (LDFA), and medial proximal tibial angle (MPTA) were calculated as previously reported (Figure 2) [24]. The HKA represents the angle formed between the mechanical axis of the femur and the mechanical axis of the tibia, denoted by (+) for valgus and (−) for varus. The LDFA represents the lateral angle between the mechanical axis of the femur and the tangential line of the femoral condyle. The MPTA represents the medial angle between the mechanical axis of the tibia and the tangent line of the tibial plateau. Moreover, knee joint line obliquity (KJLO) was measured in this study. KJLO represents the angle between the tangent line of the tibial plateau and the horizontal line, with negative values indicating medial tilt (Figure 2) [18]. In this study, the median KJLO value of the patients was −3.0. MJLO was defined as having a KJLO value ≤−3.0.

### Ultrasonographic assessment

Ultrasonographic evaluations have enabled dynamic measurements of MME under various conditions, such as in the supine and upright positions [3]. These evaluations provide a more comprehensive understanding of MME behaviour under different loading scenarios.

**Figure 3:**
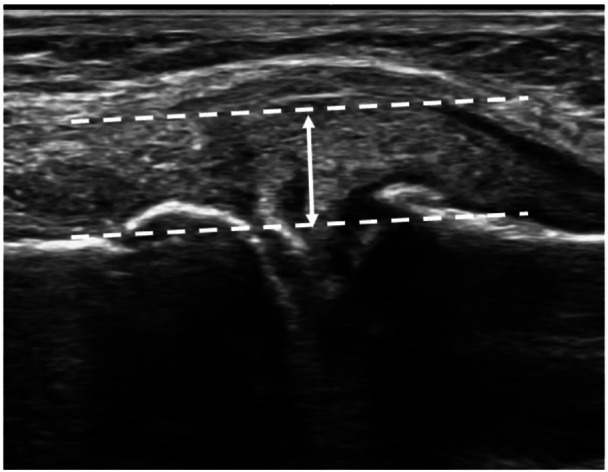
Ultrasonographic evaluation of medial meniscal extrusion. The baseline was drawn to connect the femur and the tibia without osteophytes. The target lines were drawn parallel from the baseline to the medial meniscus.

MME at knee extension in the supine and upright positions was measured using ultrasonography. All ultrasonographic examinations were performed across multiple facilities using SNiBLE (Konica Minolta, Tokyo, Japan) with an 18‐MHz linear probe. Ultrasonographic images of the medial meniscus were obtained, as previously described [3]. Briefly, images were obtained at the medial aspect of the knee using longitudinal sections parallel to the medial collateral ligament (MCL), with the femoral medial epicondyle serving as the bony landmark, as it provides the best view of the MCL in a neutral rotational position. MME was measured as the distance (mm) between the baseline, defined as the line connecting the cortex of both tibial plateaus and the bottom of the femoral medial condyle, and a target line parallel to the baseline passing through the peripheral border of the meniscal body (Figure 3). The baseline was drawn in the regions without osteophytes on both the femur and tibia and through the osteophytes to minimise bony interference for the measurement of MME. All medial meniscal assessments were performed by experienced orthopedic surgeons (K.A. and N.T.), who were blinded to MRI evaluations. This ensured unbiased and consistent ultrasonographic measurements.

### MRI evaluation

MRI was performed with mild knee flexion at each facility. Owing to the multicenter nature of this study, the MRI models and protocols were not consistent. However, all imaging procedures were performed using 1.5‐ or 3‐T MRI scanners. The PTS and medial meniscal tears were evaluated by two orthopedic surgeons (K.A. and N.T.).

**Figure 4:**
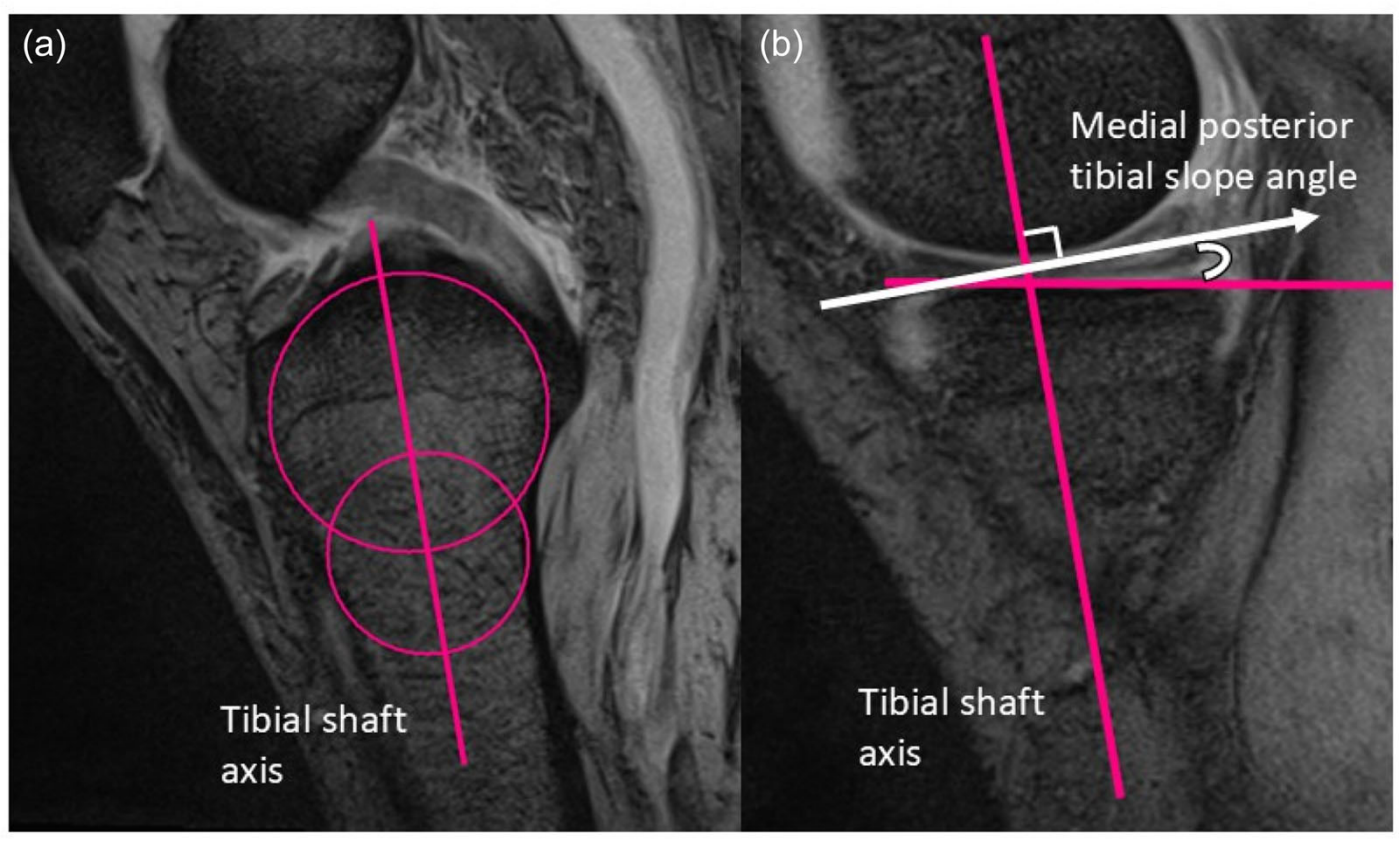
Assessment of the posterior tibial slope. (a) The tibial axis connects the centres of the two circles. (b) The medial posterior tibial slope angle is defined as the angle between the line perpendicular to the tibial axis and the line connecting the most proximal anterior and posterior subchondral bone points on the medial tibial plateau.

**Figure 5:**
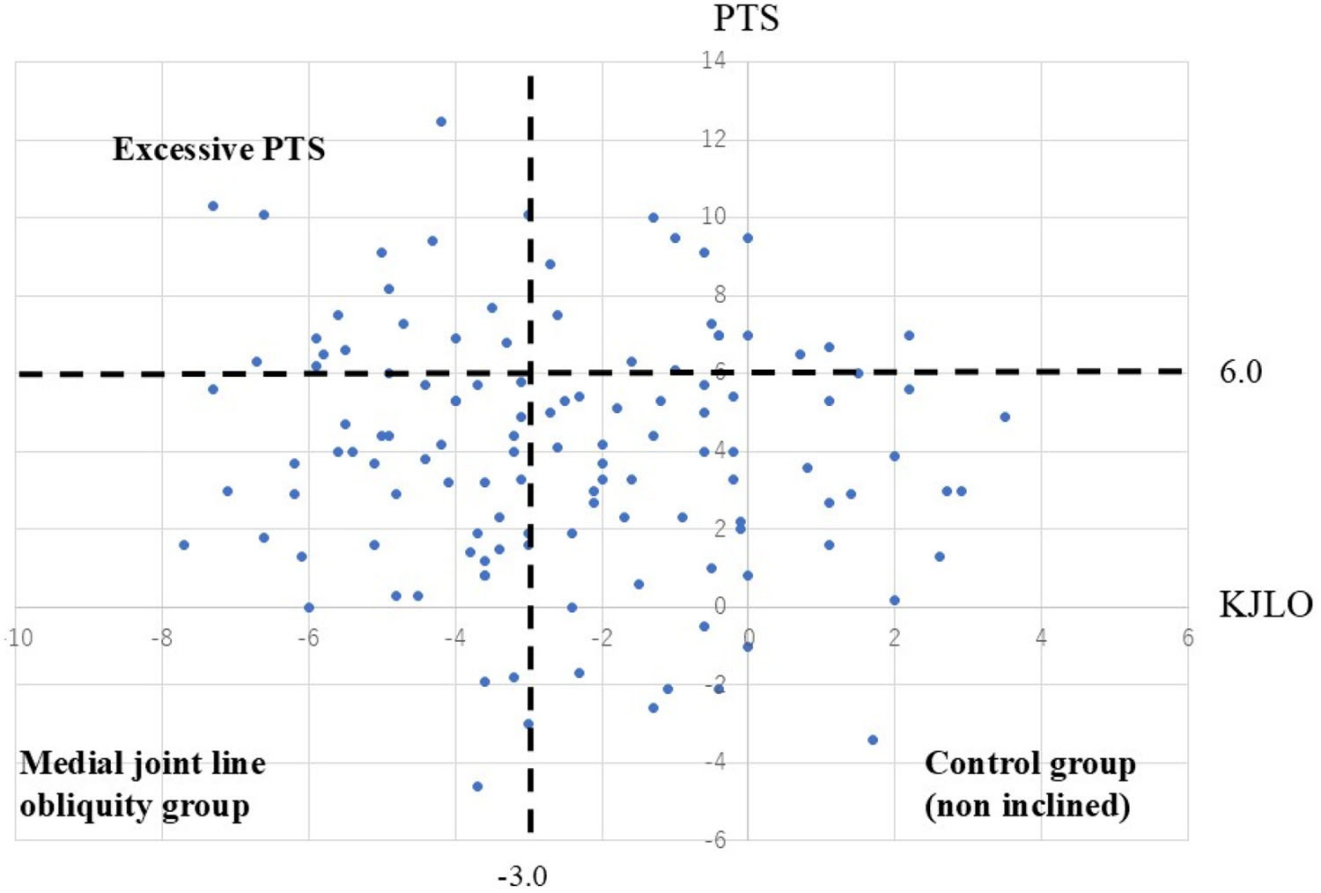
Scatter plot between KJLO and the PTS. MJLO was defined as having a KJLO ≤ − 3.0, which was the median value of KJLO. A PTS ≥ 6°, representing the 75th percentile, was defined as excessive PTS. KJLO, knee joint line obliquity; PTS, posterior tibial slope.

The medial tibial posterior slope was measured, as previously described [7]. Briefly, the central sagittal plane was identified using anatomical landmarks, such as the posterior cruciate ligament attachment and intercondylar eminence. In this plane, two circles were fitted to the anterior and posterior cortices of the tibia. The tibial axis was defined as the line connecting the centres of the circles. The medial PTS was measured as the angle between the line perpendicular to the tibial axis and the line connecting the most proximal anterior and posterior subchondral bone points on the medial tibial plateau (Figure 4). In this study, excessive PTS was defined as medial PTS ≥ 6°, which corresponded to the 75th percentile in our cohort (Figure 5), and was consistent with a previous report [7]. Although no MRI‐based definition has been established, a recent systematic review reported significantly greater medial PTS in MMPRT patients than in controls (8.1° ± 2.5° vs 4.3° ± 0.7°; *p* < 0.001) [4]. Our aim was not to determine a cutoff for predicting MMPRT, but to minimise the influence of PTS on medial meniscal injury and its contribution to MME; therefore, cases with excessive PTS were excluded using this threshold.

Medial meniscal tears were evaluated using T2/fat‐saturated proton density‐weighted MRI in both the coronal and sagittal planes. Meniscal tears were categorised as no tears, horizontal tears, flap tears (or complex tear), or MMPRT (or radial tears). MMPRT was diagnosed on the basis of the 'ghost meniscus sign' or 'cleft sign' on sagittal or coronal images [3].

### Statistical analyses

All measurements were analysed using IBM SPSS Statistics for Windows version 26.0 (IBM Corp., Armonk, NY, USA). Measurement data are expressed as means±standard deviations. The interobserver reliability of the MME measurements at knee extension in the supine and upright positions on ultrasonographic images was determined using the intraclass correlation coefficient (ICC). The ICC values for MME were *ρ* = 0.867 in the supine position and *ρ* = 0.812 in the upright position, indicating high reliability. The Shapiro–Wilk test was used to confirm data normality. The correlation between KJLO and the HKA, LDFA, MPTA, and PTS was assessed using Pearson's correlation coefficient. Continuous and categorical values were compared between the two groups using two‐sample t‐test and chi‐square test. The correlation between KJLO and MME in both the supine and upright positions was calculated. To evaluate the effect of MJLO on MME, multiple regression analysis was performed. The explanatory variables included MJLO, age, sex, body mass index, and the presence of medial meniscal injury.

G*Power version 3.1 (Heinrich‐Heine University Düsseldorf, Düsseldorf, Germany) was used for power analysis. An a priori power hoc analysis comparing MME in the supine and upright positions between the MJLO and control groups, with *α* = 0.05 and power = 0.8, showed that the required sample size was 72.

## RESULTS

The correlation between KJLO and any other morphologic features of knee is presented in Table 1. A significant correlation was found between KJLO and the LDFA (*r* = 0.48, *p* = 0.001) and MPTA (*r* = 0.41, *p* = 0.001). The patient characteristics of the MJLO and control groups are presented in Table 2. The MME values of the MJLO group in both the supine and upright positions were significantly higher than those of the control group (supine, 3.0 ± 1.0 vs. 2.4 ± 0.9 mm, *p* = 0.004; upright, 3.7 ± 1.0 vs. 3.2 ± 1.1 mm, *p* = 0.023). Meniscal tear was present in 75.6% of the MJLO group and in 75% of the control group. There were no significant differences in the types of meniscal tears between the groups, indicating a comparable distribution (*p* = 0.999). After excluding cases with excessive PTS, significant correlation was observed between KJLO and MME in the supine position (*r* = −0.21, 95% confidence interval [CI]: −0.41 to −0.01, *p* = 0.044), but not in the upright position (*r* = −0.17, 95% CI: −0.37 to 0.04, *p* = 0.105). The result of the multiple regression analysis is presented in Tables 3 and 4. MJLO (defined as KJLO ≤ − 3.0) and medial meniscus tear were independent factors for increased MME in both the supine and upright positions.

**Table 1:** Correlation between KJLO and other alignment parameters of the knee.

	*R*	95% CI	*p*‐Value
KJLO‐HKA	−0.03	−0.235 to 0.181	0.792
KJLO‐LDFA	0.48	0.334–0.605	0.001
KJLO‐MPTA	0.41	0.254–0.547	0.001
KJLO‐PTS	−0.11	−0.277 to 0.0674	0.226

*Note*: Pearson's correlation coefficient was used. The significance level was set at *p* < 0.05.

Abbreviations: HKA, hip‐knee‐ankle angle; KJLO, knee joint line obliquity; LDFA, lateral distal femoral angle; MPTA, medial proximal tibial angle; PTS, posterior tibial slope.

**Table 2:** Comparison between the MJLO and control groups.

	MJLO group (*n* = 45)	Control group (*n* = 44)	*p*‐Value
Age (years)	58.3 ± 10.5	59.8 ± 11.9	0.534
Sex (male/female)	19:26	13:31	0.271
BMI (kg/m^2^)	24.5 ± 4.5	23.0 ± 3.4	0.078
MME (mm, supine)	3.0 ± 1.0	2.4 ± 0.9	0.004
MME (mm, upright)	3.7 ± 1.0	3.2 ± 1.1	0.023
ΔMME (mm)	0.6 ± 0.4	0.7 ± 0.5	0.331
KJLO (°)	−4.5 ± 1.3	−0.4 ± 1.7	0.001
PTS (°)	2.6 ± 2.4	2.7 ± 2.3	0.823
HKA (°)	−2.5 ± 2.4	−2.9 ± 2.2	0.436
LDFA (°)	86.0 ± 1.6	87.6 ± 1.9	0.001
MPTA (°)	85.0 ± 1.7	86.3 ± 1.9	0.001
Meniscus tear			
None	11 (24.4)	11 (25)	
Horizontal	23 (51.1)	22 (50)	
Flap or complex	4 (8.9)	4 (9.1)	
Radial or MMPRT	7(15.6)	7 (15.9)	0.999

*Note*: A two‐sample t‐test or chi‐square test was used. The significance level was set at *p* < 0.05. Measurement data are expressed as means±standard deviations.

Abbreviations: BMI, body mass index; HKA, hip‐knee‐ankle angle; KJLO, knee joint line obliquity; LDFA, lateral distal femoral angle; MJLO, medial joint line obliquity; MME, medial meniscal extrusion; MMPRT, medial meniscal posterior root tear; MPTA, medial proximal tibial angle; PTS, posterior tibial slope.

**Table 3:** Multiple regression analysis of MME in the supine position.

Factor	*β*	SE	95% CI	*p*‐Value
Age	0.01	0.01	−0.01 to 0.03	0.221
Sex (0, men: 1, female)	0.21	0.21	−0.20 to 0.62	0.304
Body mass index	0.03	0.03	−0.02 to 0.09	0.186
Medial meniscus tear (0, negative: 1, positive)	0.32	0.10	0.11–0.52	0.003
MJLO (0, negative: 1, positive)	0.60	0.19	0.22–0.98	0.002

*Note*: Multiple regression analysis was performed to detect the independent factor for MME in the supine position. The significance level was set at *p* < 0.05.

Abbreviations: CI, confidence interval; MJLO, medial joint line obliquity; MME, medial meniscal extrusion; SE, standard error.

**Table 4:** Multiple regression analysis of MME in the upright position.

Factor	*β*	SE	95% CI	*p*‐Value
Age	0.01	0.01	−0.01‐0.03	0.148
Sex (0, men: 1, female)	0.11	0.22	−0.33‐0.55	0.624
Body mass index	0.03	0.03	−0.03‐0.09	0.283
Medial meniscus tear (0, negative: 1, positive)	0.38	0.11	0.16‐0.61	0.001
MJLO (0, negative: 1, positive)	0.50	0.21	0.09‐0.91	0.017

*Note*: Multiple regression analysis was performed to detect the independent factor for MME in upright position. The significance level was set at *p* < 0.05.

Abbreviations: CI, confidence interval; MJLO, medial joint line obliquity; MME, medial meniscal extrusion; SE, standard error.

## DISCUSSION

The most important finding in this study was the correlation of MJLO and MME in both the supine and upright positions. KJLO was associated with the LDFA and MPTA, but not with the HKA or PTS. These findings suggest that, when evaluating lower limb alignment, assessing KJLO in the standing position is important for understanding load distribution within the knee joint, in addition to assessing varus or valgus alignment using the HKA.

In this study, MJLO significantly increased MME in both the supine and upright positions. However, the frequency of medial meniscal tear was not significantly different in the two groups. The key to understanding these results has been explained in previous studies. A biomechanical study by Wang et al. demonstrated that medially directed shearing force caused by a medial downslope increased the contact pressure on the middle segment of the medial meniscus, with the highest pressure observed at a 7° inclination [22]. Moreover, a higher MJLO could promote MME by shifting the medial femoral condyle contact point on the medial tibial plateau medially, generating an outward force to the medial meniscus. In contrast, KJLO showed only a weak correlation with MME in supine position and no correlation with MME in upright position in this study. Several factors may have masked the correlation between KJLO and MME. At early‐stage KOA, the hoop mechanism of the meniscus might remain functional in many cases and prevent a significant increase in MME until the inclination exceeds a certain threshold. Furthermore, an increase in MME from the supine to the upright position, namely dynamic MME, has been observed in normal knees; however, this can be affected by MMPRT or degenerative meniscal tears, which may act as confounding factors [8]. On the other hand, in the multiple regression analysis with the threshold set at a 3° inclination, MJLO was identified as a significant factor in both upright and supine positions. These results suggested that when the medial inclination exceeds the threshold, the increased contact pressure on the medial meniscus contributes to MME increase.

Varus alignment has been reported to increase contact pressure on the medial meniscus, which can directly contribute to an increase in MME [23]. Moreover, it could also increase the risk of MMPRT, which in turn increases the chances of MME [17]. However, in this study, varus alignment was not a significant factor affecting MME in either the supine of the upright position. This may be because the participants had early‐stage KOA with only mild varus alignment, resulting in limited influence on MME. There have been clinical studies on lower limb alignment [2, 14, 15, 16]. Nakayama et al. showed that the presence of a varus deformity negatively affected the healing rate of meniscal injuries [15]. Nakamura et al. reported that femoral‐varus tibial‐valgus osteotomy for neutrally aligned KOA with severe KJLO presented good clinical outcomes with modified KJLO [14]. Although the cutoff value for postoperative KJLO has been evaluated in some studies [2, 16], there was no study to define the preoperative cutoff value of KJLO for predicting poor outcomes in early‐stage KOA. In the present study, the median value of KJLO was used as the cutoff value. The definition of the preoperative cutoff value of KJLO was necessary in the future. The results of this study suggested that MJLO significantly affects MME and that joint line orientation should be considered in addition to varus/valgus alignment assessed.

In this study, the presence of medial meniscus tear was an independent risk factor of larger MME in both the supine and upright positions. Previous studies have shown that MMPRT increases MME [3, 8, 21]. Chiba et al. found that 5‐ and 7‐mm MMEs on ultrasonographic findings were the optimal cutoff values for both early‐stage (adjusted odds ratio, 6.280) and progressive OAs (adjusted odds ratio, 15.003) [3]. Moreover, not only MMPRT but also degenerated horizontal meniscus tear was associated with large MME [1]. In this study, there was no significant difference in the presence of meniscal tear between the two groups. Moreover, the two groups had almost the same ratio of meniscus tear. Therefore, consistent with previous reports, meniscal tear was an independent factor for MME in both the supine and upright positions in this study; however, its contribution to the difference in MME between the two groups was estimated to be limited.

Previous studies have reported that a higher PTS is associated with a high frequency of MMPRT or radial tear, and such cases present with large MME [4, 10]. A recent systematic review found that the PTS in the MMPRT group was significantly greater than that in the control group (8.1° ± 2.5° vs. 4.3° ± 0.7°, *p* < 0.001) [4]. Thus, to minimise the influence of the PTS, cases with excessive PTS were excluded from this study.

An ultrasonographic device was used to evaluate MME values in this study. The dynamic evaluation of MME between the upright and supine positions can be performed using ultrasonography. Previous studies have shown a significant correlation between ultrasonographic evaluation and MRI findings in MME (*r* = 0.800–0.865) [1, 10, 20]. Karpinski et al. reported that dynamic changes in the MME values between the supine and upright positions were absent in the MMPRT because of complete loss of hoop function [8]. In MMPRT cases, dynamic MME is expected to disappear; however, the period between injury and examination may influence MME. This study was limited by the unknown period between injury and examination. However, the ratios of MMPRT or radial tears were 15.6% in the MJLO group and 15.9% in the control group. Moreover, there was no significant differences in ΔMME between the two groups. Therefore, there might be significant difference in the condition of hoop function of the medial meniscus between the two groups, and the difference in MME may be partly attributable to the shearing force caused by MJLO.

This study has some limitations. First, because this was a multicenter study, variations in MRI protocols and ultrasound image quality across sites were unavoidable. Therefore, monthly meetings were conducted to confirm the validity of the ultrasound images, and the findings were evaluated by a single surgeon (K.A.). Second, MME evaluations were limited to the supine and upright positions. As no evaluation was performed during single‐leg standing or knee flexion with loading, further investigation is necessary to characterise the MME features in each slope group. Third, there were no data on healthy controls; therefore, the baseline of MME was unknown. As the participants of this study were patients with early‐stage KOA, the MME in the non‐inclined group was still abnormal. To determine the impact of MJLO, further studies with a healthy control group are needed. Finally, this study lacked longitudinal data. Differences in MME were observed between the MJLO and control groups in this study. However, the clinical outcomes influenced by these differences remain unclear. Long‐term studies are essential to determine the optimal surgical timing and techniques.

Based on the present results, MJLO appears to contribute to an increase in MME, suggesting higher contact pressure on the medial meniscus in both the supine and upright positions in early‐stage KOA. In particular, the risk of MME is elevated in cases of MJLO accompanied by a medial meniscal tear. In such cases, a simple meniscal repair procedure alone may cause poor outcome; therefore, careful consideration is required for surgical planning in cases of KJLO.

## CONCLUSION

MJLO and medial meniscus tear may be risk factors for medial meniscal extrusion in both the supine and upright positions in early‐stage knee osteoarthritis.

## AUTHOR CONTRIBUTIONS

*Conceptualisation*: Kazuki Asai and Junsuke Nakase. *Data curation*: Tomoyuki Kanayama, Naoki Takemoto, and Manase Nishimura. *Formal analysis*: Kazuki Asai. *Investigation*: Kazuki Asai, Junsuke Nakase, Yosuke Shima, Kenichi Goshima, Kazunari Kuroda, Takeshi Oshima, Yasushi Takata, Mitsuhiro Kimura, Kengo Shimozaki, and Kentaro Fujita. *Methodology*: Kazuki Asai and Naoki Takemoto. *Project administration*: Junsuke Nakase. *Supervision*: Satoru Demura. *Original draft writing*: Kazuki Asai. *Review and editing*: Junsuke Nakase.

## CONFLICT OF INTEREST STATEMENT

The authors declare no conflict of interest.

## ETHICS STATEMENT

This study was approved by the ethics committee of Kanazawa university hospital (no. 2328). The written informed consent was obtained from all the patients.

## Data Availability

Data available on request from the authors.
